# Factors Affecting Sperm Quality Before and After Mating of Calopterygid Damselflies

**DOI:** 10.1371/journal.pone.0009904

**Published:** 2010-03-26

**Authors:** Kaori Tsuchiya, Fumio Hayashi

**Affiliations:** Department of Biology, Tokyo Metropolitan University, Tokyo, Japan; University of Exeter, United Kingdom

## Abstract

Damselflies (Odonata: Zygoptera) have a more complex sperm transfer system than other internally ejaculating insects. Males translocate sperm from the internal reproductive organs to the specific sperm vesicles, a small cavity on the body surface, and then transfer them into the female. To examine how the additional steps of sperm transfer contribute to decreases in sperm quality, we assessed sperm viability (the proportion of live sperm) at each stage of mating and after different storage times in male and female reproductive organs in two damselfly species, *Mnais pruinosa* and *Calopteryx cornelia*. Viability of stored sperm in females was lower than that of male stores even just after copulation. Male sperm vesicles were not equipped to maintain sperm quality for longer periods than the internal reproductive organs. However, the sperm vesicles were only used for short-term storage; therefore, this process appeared unlikely to reduce sperm viability when transferred to the female. Males remove rival sperm prior to transfer of their own ejaculate using a peculiar-shaped aedeagus, but sperm removal by males is not always complete. Thus, dilution occurs between newly received sperm and aged sperm already stored in the female, causing lower viability of sperm inside the female than that of sperm transferred by males. If females do not remate, sperm viability gradually decreases with the duration of storage. Frequent mating of females may therefore contribute to the maintenance of high sperm quality.

## Introduction

**Figure 1:**
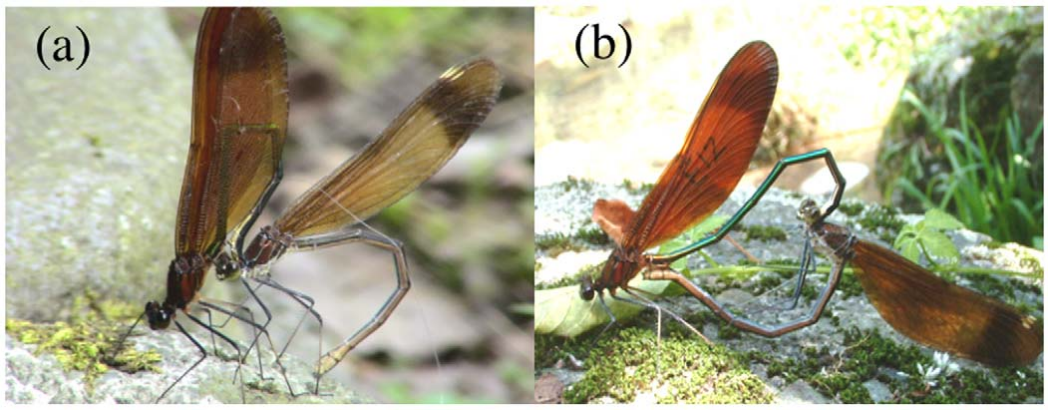
Mating of *Calopteryx cornelia*. Individuals with a pterostigma are females. (a) Sperm translocation by the male from the opening of the internal reproductive system to the sperm vesicles under abdominal segments 2 and 3. (b) After sperm translocation, the male inserts the aedeagus under abdominal segments 2 and 3 into the female genital opening to remove rival sperm stored in the female storage organs. After removal, the male transfers sperm from the sperm vesicles to the female storage organs along a slit of the aedeagus.

Males usually possess specialised storage organs in which mature sperm are maintained between spermatogenesis and copulation. The females of many species with internal fertilisation also possess storage organs to house sperm between mating and egg fertilisation. In most insect species, seminal vesicles and spermathecae are the sperm storage organs of males and females, respectively, and sperm are transferred directly to the female [1]. However, damselflies (suborder Zygoptera) exhibit a complex system of sperm transfer and storage [2]. Sperm produced in the testes are transferred through the vas deferens to the seminal vesicles (SEV) for storage until mating. At mating, once the male grasps the female head region with his abdominal terminalia, he translocates sperm stored in the SEV from the opening of the internal reproductive system, positioned near the abdominal tip, into the sperm vesicles (SPV) of the secondary genitalia under abdominal segments 2 and 3 (Fig. 1a). After completing sperm translocation, the aedeagus of the secondary genitalia is inserted into the female genital opening, and sperm in the SPV are transferred along a slit of the aedeagus into the female storage organs (Fig. 1b). Females usually store the received sperm in two storage organs, the bursa copulatrix (BC) and the spermatheca (SP).

However, not all sperm survive in the storage organs [e.g., [3]–[5]]. Sperm age after spermatogenesis, and the duration of sperm storage affects sperm quality through the aging process and chemical/physical damage to the cells [6], [7]. Furthermore, sperm can also be damaged during transport between storage organs. Because additional steps of sperm transfer lead to a greater chance of sperm damage, the complex system of sperm transfer in damselflies may reduce sperm quality more severely than direct ejaculation into the female storage organ. In this study, we assessed the viability of stored sperm in male and female sperm-storage organs at each stage of copulation in two damselfly species of the family Calopterygidae, *Mnais pruinosa* and *Calopteryx cornelia*. In particular, we assessed the ability of these species to store sperm in the SPV, the peculiar organ of damselflies. Our experiments provided a unique opportunity to examine how each storage organ involved in the mating process is equipped to protect against sperm damage and to discuss the stepwise changes in sperm quality during insect mating.

*Mnais pruinosa* and *C. cornelia* coexist in a wide part of Japan, but the two species differ in the pattern of adult emergence: the former exhibits synchronous emergence, whereas the latter emerges continuously [8]. Thus, the age structures of adult populations differ between the two species [9]–[12]. In insects, the quality of male-stored sperm is affected by male age [13]–[15]. In turn, different age structures of mating populations may affect the quality of sperm available for females. In this study, we examined the effect of age on male-stored sperm and compared seasonal patterns of sperm quality in males and females between the two species.

Sperm quality was assessed in terms of viability using fluorescent staining of live and dead sperm. This method has been increasingly used to examine the causes and consequences of variation in sperm quality [16]. When using this method, the percentages of live and dead sperm may be affected by the dilution of sperm in artificial buffer, such that the obtained values represent relative, and not absolute, sperm viability and likely indicate the robustness of sperm when exposed to the buffer [5], [16]. Thus, we use the term “sperm robustness” for this measure.

## Materials and Methods

### Seasonal Patterns of Sperm Robustness

From 15 May to 14 July 2002, 35 males and 21 females of *M. pruinosa* were collected from the Kobotoke River, a tributary of the Tama-gawa River in Tokyo, central Japan. The adult flying season of this species is restricted to early summer, and three seasonal groups were distinguished: early (15 to 22 May, during which newly-emerged males were collected [17]), middle (28 May to 8 June), and late periods (17 June to 14 July).

On the day of collection, the head and thoracic region of *M. pruinosa* were placed into a glass vial (22 mm in diameter, 50 mm in height), which was then filled with CO_2_ gas for anesthetisation. Using this method, the abdominal region was not directly anesthetised. In males, paired SEVs were then dissected out and placed in 50 µL of Grace's insect cell culture medium (Gibco BRL, Life Technologies, New York, NY, USA). In females, sperm storage organs (BC, SP) were dissected out and placed in 50 µL of Grace's buffer. The SP was immediately separated from the BC using fine forceps and then placed in an additional 50 µL of Grace's buffer. Sperm were mixed in the buffer by gentle pipetting.

Sperm robustness was assessed using a SYBR-14:propidium iodide live/dead sperm viability kit (Molecular Probes, Eugene, OR, USA). A 10-µL sperm suspension, 10 µL of a 0.048 mmol/L propidium iodide solution (2.4 mmol/L propidium iodide solution diluted 50× with Grace's buffer), and 1 µL of a 0.004 mmol/L SYBR-14 solution (1 mmol/L SYBR-14 diluted by 250× with dimethyl sulfoxide) were mixed in a 500-µL tube and incubated in the dark for 5 min at room temperature. Subsequently, 8 µL of this solution was dropped onto a slide glass and covered with a 24×32-mm cover glass. Normal (auto exposure) and fluorescence photographs (8-s exposure via excitation filter of 470–490 nm; BP470–490; Olympus, Tokyo, Japan) were taken at the same angles under a fluorescence microscope (200×, IX70; Olympus). Observations ended within 20 min of the start of incubation. Using this sperm viability kit, live and dead sperm were stained green and red, respectively. However, four color groups were actually distinguishable: (i) no fluorescence; (ii) entirely green; (iii) green with some red spots; and (iv) entirely red in the head region. Non-fluorescing sperm moved their flagella (i.e., they were alive), and sperm that were green with red spots had possibly been injured in the staining solution [18]; thus, only entirely red sperm were counted as non-viable, and all others were considered viable. The percentage of viable sperm was calculated based on the mean of 84 sperm (*n* = 63, SE = 7) per individual preparation. Sampling of damselflies usually occurred from 11:00 to 15:00 in the field. Subsequent anesthetisation, dissection, and sperm viability measurements were conducted in the laboratory from 17:00 to 24:00 on that same day.

Sperm suspensions, which had been preserved at −20°C, were diluted 1, 50, or 500× according to their apparent concentrations. The total number of sperm in a given tube was estimated by counting all sperm in 1 µL. After vortexing, 1 µL of the sperm suspension was dropped onto a slide glass, dried at room temperature for several days, fixed in pure ethanol for 10 min, dried again for about 20 min, placed in 5% Giemsa solution for 30 min, washed in distilled water for 5 min, covered with a cover glass, and observed under a compound light microscope (100×; BH-2; Olympus). Counts of sperm number from five different 1-µL drops per tube were averaged.

From 14 June to 6 September 2003, 40 males and 24 females of *C. cornelia* were collected from the Kobotoke and Yozawa Rivers, both of which are tributaries (10 km distant) of the Tama-gawa River. The adult flying season of this species occurs from early summer to early autumn, and three seasonal groups were distinguished: early (14 to 30 June 2003), middle (16 to 31 July 2003), and late periods (22 August to 6 September 2003). On the day of collection, sperm storage organs were dissected out after anesthetising in a CO_2_-filled glass vial (25 mm in diameter, 60 mm in height), and data for sperm number and robustness were collected using the same method as for *M. pruinosa*. The percentage of viable sperm was calculated based on the mean of 228 sperm (*n* = 87, SE = 11) per individual preparation.

### Age-Related Sperm Robustness in Males

From 30 April to 26 June 2005 at the Kobotoke River, 318 young males of *M. pruinosa* were captured, marked with individual numbers on the forewings using paint markers, and then immediately released at the site of capture. Thirty-four males were recaptured, and their SEVs were dissected out to estimate sperm number and robustness using the same method as for observations of seasonal patterns. Male age at first capture was estimated from the color of the forewing pterostigma. Field-captured newly emerged males, which have a white pterostigma (*n* = 8), were kept individually in plastic cages (100 mm in diameter, 110 mm in height) at 23±1°C with a photoperiod of 14 h light: 10 h dark. All inner surfaces of the cages were covered with fine nylon netting, and a piece of wet filter paper was placed on the bottom to prevent desiccation. Males were fed five cultured adult *Drosophila melanogaster* every day by transferring individual flies into their mouthparts using forceps. Water was also provided daily with forceps until the damselflies ceased drinking. Median times for color changes of the pterostigma were as follows: 1 day to change from white to slightly yellow, 2 days to turn yellow, 3 days to pink, 7 days to pale red, 10 days to red, and 13 days to dark red. Thus, male age was calculated as the age at first capture (i.e., estimated days from adult eclosion) + the duration (days) between release and recapture.

From 2 May to 10 August 2005 at the Yozawa and Kobotoke Rivers, 223 males of newly emerged *C. cornelia* (the ventral side of the thoracic region and the ventral side of the abdominal tip were yellow) were captured and released at the capture site after marking using the same method as for *M. pruinosa*. If recaptured (37 males), SEVs were dissected out, and the number and robustness of sperm were estimated.

### Sperm Robustness Before and After Copulation

From 27 May to 7 July 2004, seven and eight mature females of *M. pruinosa* and *C. cornelia*, respectively, were each mated with a different male using the hand-pairing method at the Yozawa River [19]. All pairs were dissected on the day of pairing, and sperm number and robustness in the SEV, BC, and SP were estimated.

### Sperm Storage in the SEV and SPV

From 1 June to 10 July 2005, 18 mature males of *M. pruinosa* were collected at the Yozawa River. These males were then mated with mature females using the hand-pairing method, but the copulation was interrupted just before their own ejaculation [19]. Therefore, these males stored sperm in both the SEV and SPV. The males were then reared using the same method described above and were dissected on the first (*n* = 4) or third (*n* = 14) day after collection to examine temporal changes in the number and robustness of sperm stored in these two organs.

In total, 18 mature males of *C. cornelia* were collected at the Yozawa River from 28 July to 9 August 2004, 15 to 21 July 2005, and 3 to 10 August 2006. Their copulation was interrupted (as described above), and the number and viability of sperm in the SEV and SPV were examined on the first (*n* = 12) and third (*n* = 6) days after rearing. Rearing conditions were the same as for *M. pruinosa*, except that *C. cornelia* were fed nine *Drosophila* per day, as this species is larger than *M. pruinosa*.

### Statistical Analyses

All summary statistics are described as means±1 SE and were analysed using t-tests, paired sample t-tests, analyses of variance (ANOVA), and correlation coefficients (*r*). Because variances were not equal for t-tests, we used Welch's approximate t [20]. The percentage of viable sperm was arcsine-transformed for all statistical analyses to satisfy the assumption of normality [20].

## Results

### Seasonal Patterns of Sperm Robustness

**Figure 2:**
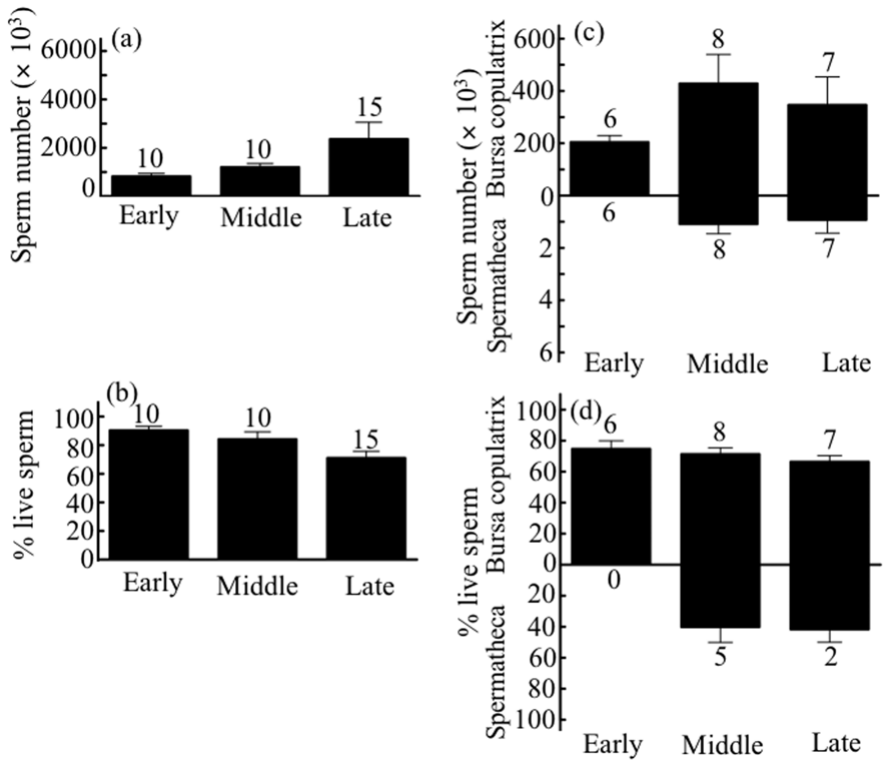
Seasonal patterns (early, middle, and late reproductive seasons) of the number and robustness of stored sperm in male (a, b) and female (c, d) *Mnais pruinosa*. Bars indicate +SE, with the number of individuals examined.

In *M. pruinosa*, all dissected males contained sperm in the SEV. The mean number of sperm tended to increase with season, but the pattern was not significant (ANOVA: F_2, 32_ = 2.45, *p* = 0.10; Fig. 2a). In contrast, sperm robustness significantly decreased with season (ANOVA: F_2, 32_ = 6.13, *p* = 0.006; Fig. 2b). All dissected females also contained sperm in their sperm storage organs. The number of sperm in the BC and SP did not differ among the three seasonal periods (ANOVA: F_2, 18_ = 1.31, *p* = 0.30 for the BC; F_2, 18_ = 2.18, *p* = 0.14 for the SP; Fig. 2c). Similarly, the robustness of sperm in the BC and SP did not differ across seasons (ANOVA: F_2, 18_ = 0.95, *p* = 0.40 for the BC; t-test: t_5_ = 0.13, *p* = 0.90 for the SP; Fig. 2d), although the number of sperm in the SP was much lower than in the BC; thus, in most cases, we could not estimate the proportion of live sperm in the SP.

**Figure 3:**
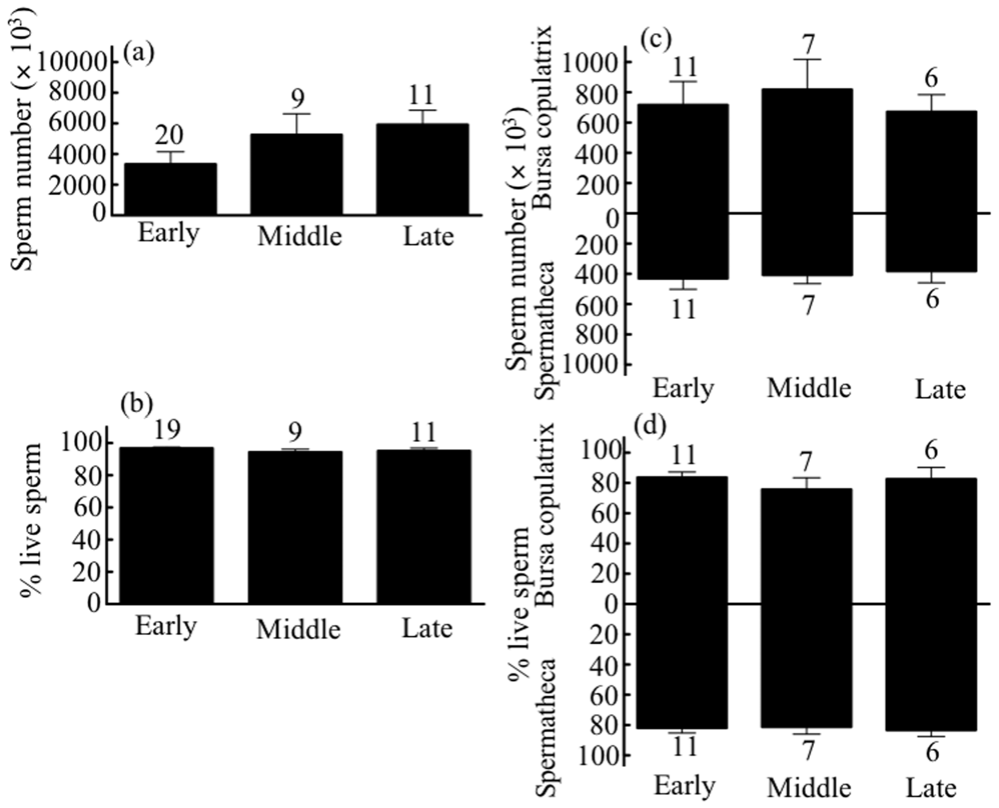
Seasonal patterns (early, middle, and late reproductive seasons) of the number and robustness of stored sperm in male (a, b) and female (c, d) *Calopteryx cornelia*. Bars indicate +SE, with the number of individuals examined.

In male *C. cornelia*, the mean number of sperm in the SEV tended to increase with season, but the pattern was not significant (ANOVA: F_2, 37_ = 2.07, *p* = 0.14; Fig. 3a). Sperm robustness did not differ among the three seasonal periods (F_2, 36_ = 0.45, *p* = 0.64; Fig. 3b). Similarly, in females, seasonal changes were not detected in sperm number (ANOVA: F_2, 21_ = 0.18, *p* = 0.84 for the BC; F_2, 21_ = 0.60, *p* = 0.56 for the SP; Fig. 3c) or robustness (F_2, 21_ = 0.29, *p* = 0.75 for the BC; F_2, 21_ = 0.03, *p* = 0.97 for the SP; Fig. 3d).

### Age-Related Sperm Robustness in Males

**Figure 4:**
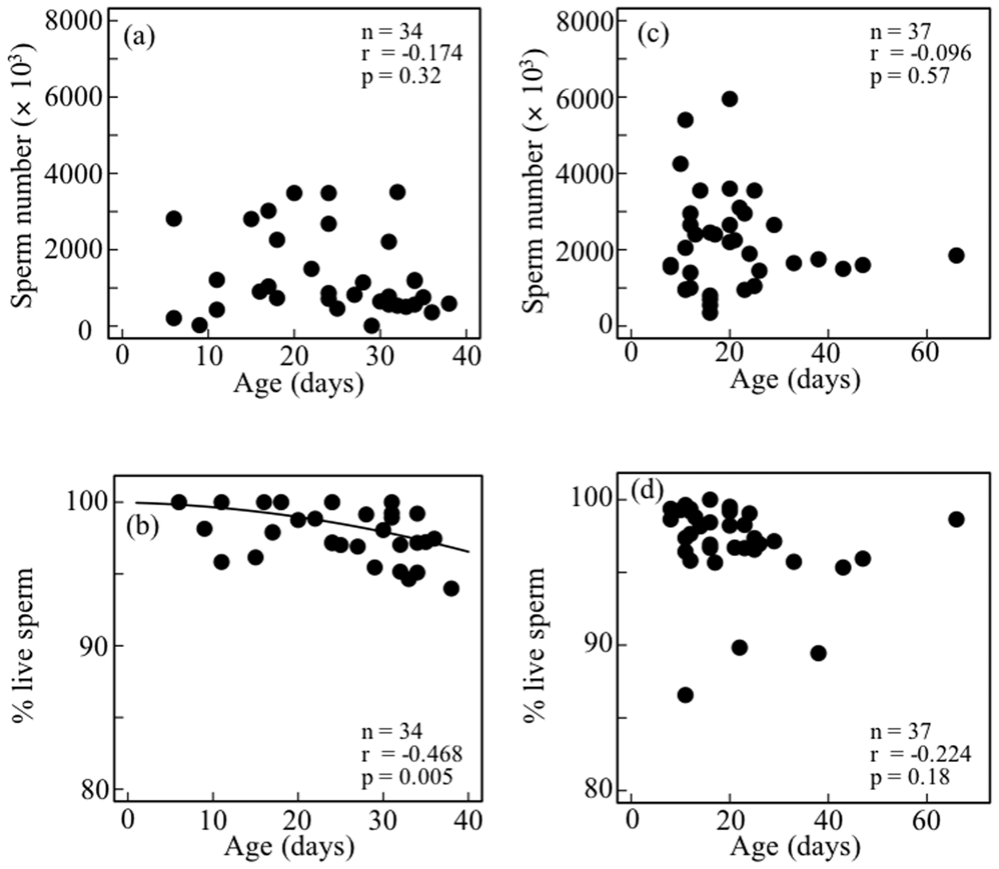
Effects of age on the number and robustness of sperm stored in the seminal vesicles of male *Mnais pruinosa* (a, b) and *Calopteryx cornelia* (c, d). The regression line for arcsine-transformed data is shown.

The number of sperm stored in the SEV was not clearly related to male age in either *M. pruinosa* (Fig. 4a) or *C. cornelia* (Fig. 4c). However, the proportion of viable sperm in the SEV decreased significantly with male age in *M. pruinosa* (Fig. 4b), but not in *C. cornelia* (Fig. 4d).

### Sperm Robustness Before and After Copulation

**Figure 5:**
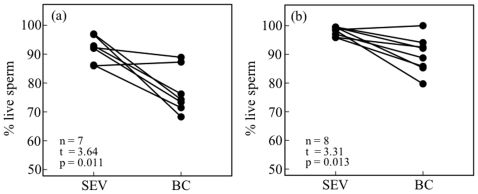
Robustness of sperm in male seminal vesicles (SEV) and the female bursa copulatrix (BC) just after copulation of pairs (connected with lines) of *Mnais pruinosa* (a) and *Calopteryx cornelia* (b). Results of paired t-tests are shown.

The mean numbers of sperm remaining in the male SEV and those contained in the female BC and SP in mated pairs of *M. pruinosa* were 709,000 (*n* = 7, SE = 139,000), 110,000 (*n* = 6, SE = 27,000), and 1700 (*n* = 6, SE = 800), respectively. The proportion of live sperm was significantly higher in the SEV (91.9±1.7%, *n* = 7) than in the BC (77.1±3.0%, *n* = 7; Fig. 5a). Only three females contained small amounts of sperm in the SP; thus, sperm robustness could not be estimated for this organ. For pairs of *C. cornelia*, the mean numbers of sperm in the SEV, BC, and SP were 2,944,000 (*n* = 8, SE = 685,000), 510,000 (*n* = 8, SE = 101,000), and 262,000 (*n* = 8, SE = 40,000), respectively. The mean proportions of live sperm in the SEV, BC, and SP were 98.0±0.5%, 89.8±2.2%, and 89.4±2.0%, respectively. The proportion of live sperm did not differ between the BC and SP (paired t-test, t = 0.16, *p* = 0.88) but was significantly different between the SEV and BC (Fig. 5b). Thus, in both species, the proportion of live sperm decreased when transferred from males to females.

### Sperm Storage in the SEV and SPV

**Figure 6:**
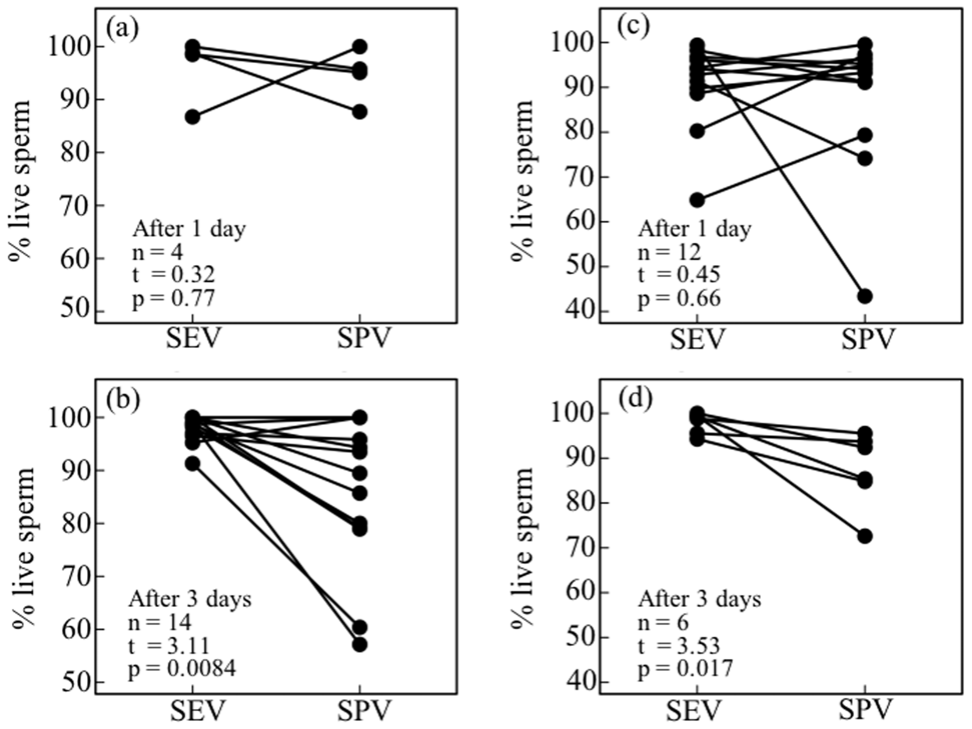
Robustness of stored sperm in the seminal (SEV) and sperm (SPV) vesicles 1 and 3 days after copulation in *Mnais pruinosa* (a, b) and *Calopteryx cornelia* (c, d). Data from the same individual are connected by lines. Results of paired t-tests are shown.

In *M. pruinosa*, the numbers of sperm present 1 day after copulation were 684,000 (*n* = 4, SE = 127000) in the SEV and 38,400 (*n* = 4, SE = 20,000) in the SPV; the numbers of sperm present 3 days after copulation were 960,000 (*n* = 12, SE = 172,000) in the SEV and 16,200 (*n* = 12, SE = 5300) in the SPV. The number of sperm present 1 and 3 days after copulation did not differ significantly in the SEV (t_14_ = 0.88, *p* = 0.39) or the SPV (t_14_ = 1.06, *p* = 0.36). Using the combined data, the proportion of sperm transferred from the SEV to SPV at copulation was estimated to be 8.4% (*n* = 16, SE = 4.2). Sperm robustness did not differ between the SEV and SPV 1 day after copulation (Fig. 6a), but decreased significantly in the SPV after 3 days (Fig. 6b).

In *C. cornelia*, the numbers of sperm present 1 day after copulation were 2,493,000 (*n* = 12, SE = 553,000) in the SEV and 1,469,000 (*n* = 12, SE = 213,000) in the SPV; the numbers of sperm present 3 days after copulation were 1,404,000 (*n* = 6, SE = 449,000) in the SEV and 531,000 (*n* = 6, SE = 144,000) in the SPV. The number of sperm present 1 and 3 days after copulation did not differ in the SEV (t_16_ = 1.28, *p* = 0.22), but did differ in the SPV (t_16_ = 2.92, *p* = 0.010). When data for 1 day after copulation were used, the proportion of sperm transferred from the SEV to SPV at copulation was estimated to be 32.5% (*n* = 12, SE = 10.2). Sperm robustness did not differ between the SEV and SPV 1 day after copulation (Fig. 6c), but decreased significantly in the SPV after 3 days (Fig. 6d).

## Discussion

### Sperm Quality Before and After Mating

The proportion of live sperm in the female storage organs was lower than that of male stores even just after copulation. Male damselflies have an SPV, which they use to transfer sperm. Additional steps of sperm transfer may cause accumulation of sperm damage and ultimately decrease sperm quality more severely than simple and direct ejaculation from the male reserves to the female storage organs. We observed that the robustness of sperm in the SPV greatly decreased after 3 days of storage, suggesting that the SPV does not store sperm for long periods of time. However, it has been suggested that all sperm in the SPV are transferred to the female during the final stage of natural copulation [21]. Furthermore, the total duration of copulation is only 91 s in *M. pruinosa* and 254 s in *C. cornelia* [19]. Thus, the SPV is only used for short-term storage of sperm. Thus, this pathway is unlikely to reduce sperm viability to the level observed just after transfer to the female. Not all of the sperm stored in the SEV was used during a single mating; this strategy allows males to mate multiple times even during a short period of time. However, the proportion of sperm used for a single copulation differed between the two species (i.e., 8.4% in *M. pruinosa* and 32.5% in *C. cornelia*). Thus, the turnover between sperm production in the testes and consumption at mating was much higher in *C. cornelia* than in *M. pruinosa*.

Another factor causing reductions in sperm quality after mating may be incomplete sperm removal by males. Male calopterygid damselflies can remove sperm stored in the female sperm storage organs before depositing their own ejaculates [22], thus increasing their fertilisation success [23]. Males develop both an aedeagus with a recurved head, which is used to gather sperm in the BC within its cavity, and paired, spiny lateral processes, which are used to remove sperm from the SP [22]. The volume of sperm removed can reach as high as 100% in several species [24]. In *M. pruinosa* and *C. cornelia*, males can also remove sperm [19]. However, the female sperm-storage organs of the two species differ in shape and function. Female *C. cornelia* have smaller BCs and larger SPs compared with female *M. pruinosa* [25]. The former species allocates received sperm nearly equally between the BC and the SP, and sperm exhibit similar survival rates in the two organs. However, the proportion of live sperm gradually decreases with the duration of storage: i.e., from 81% to 70% after 5 days in the BC and from 83% to 74% after 5 days in the SP [26]. In contrast, *M. pruinosa* primarily stores sperm in the BC, because the SP is typically empty of sperm and is less able to maintain sperm quality than the BC [26]. The proportion of live sperm decreases from 71% to 63% after 5 days in the BC but is always lower (41% to 38%) in the SP [26]. In *M. pruinosa*, the volume of sperm removed by males increases with the duration of copulation [27], such that removal is not complete for shorter copulations. In *C. cornelia*, BC sperm are usually removed completely, but SP sperm often remain [19]. Because sperm in the BC and SP are mixed [27], dilution occurs between newly received sperm and aged sperm already stored in the female, resulting in lower viability of the sperm within the female than in sperm transferred by males.

Sperm deteriorate with age, and older sperm can negatively affect zygote viability [6], [7]. Thus, natural selection is expected to favour females that prevent fertilisation by aged sperm. Remating avoids fertilisation with aged sperm by increasing the amount of young sperm in the female stock [6]. Similarly, if female *M. pruinosa* and *C. cornelia* do not remate, sperm viability would decrease with the time spent in the female storage organs [26]. Frequent mating of females may therefore contribute to the maintenance of high sperm quality, as males remove sperm stored in the female before transferring their own sperm, although removal is not always complete.

### Seasonal Patterns in Sperm Quality

*Mnais pruinosa* exhibits a synchronous emergence pattern [9], [10]. Younger individuals are abundant during the early adult flying period, but spent individuals increase during the late period. The number of sperm stored in the male SEV can be represented as the balance between rates of sperm production (in the testes), sperm mortality (in the SEV), and consumption at mating. If males do not mate, sperm stocks may gradually increase. Based on our observations, however, the amount of stored sperm did not significantly increase with season (Fig. 2a) or with age (Fig. 4a). These patterns may have been caused in part by large differences in mating rates among individual males [28]. In contrast, sperm robustness depended on both season (Fig. 2b) and male age (Fig. 4b). The robustness of stored sperm in the SEV was lower in older males; therefore, the mean population viability was much lower during the late reproductive season. In contrast, *C. cornelia* has a longer emergence period than does *M. pruinosa* [11], [12]. Emergence occurs continuously for about 5 months, such that young and spent individuals intermix during the adult flying season. The number of sperm stored in the SEV was not affected by season (Fig. 3a) or male age (Fig. 4c). Similarly, the robustness of stored sperm did not change with season or age (Fig. 3b, 4d). In crickets, sperm quality reportedly increases with male age [29]. The reasons why we observed conflicting results between species for the effect of age on sperm robustness remain unclear.

In *M. pruinosa*, the average viability of sperm in males was lowest in the late reproductive period, due to an increase in the number of old males during this period. However, females maintained sperm viability even during this late period, suggesting the possibility of non-random mating based on sperm quality. This strategy may involve passive selection by females through age-related male mating ability and/or active mate choice by females themselves. Male *M. pruinosa* employ three types of mate-securing tactics: territorial, sneaky, and opportunistic [27], [28], [30]. Territorial males secure matings with females that fly into, or through, their territory. Male-defended patches are used for females to lay eggs. Sneaky males usually perch in or near areas of a territory, awaiting the chance to copulate with visiting females. Opportunistic males secure matings with females either at riverside feeding sites or while traveling between feeding sites and the stream. However, the relationship between male mating tactics and sperm quality has not yet been examined. In a study of male–male territorial combats in *M. costalis*, a closely related species to *M. pruinosa*, the outcome was determined by the age-related fat reserves of males [31]. Declines in sperm viability are also related to age in *M. pruinosa*. If age-related changes in physiological condition can affect the mating success of males, females are expected to mate with males with higher sperm quality. Determining whether females mate on the basis of the value of territory or the male phenotype is often difficult [32]. Females may select males directly, based on their wing pigmentation [32], [33], and/or indirectly, via a preference for oviposition sites [34], [35]. Future studies should focus on which process best maintains high quality sperm in damselfly females.
